# Supplementary material for: Within and between-day variation and associations of symptoms in Long Covid: Intensive longitudinal study
Source: PLoS One. 2023 Jan 19;18(1):e0280343. doi: 10.1371/journal.pone.0280343 (PMC9851560; doi:10.1371/journal.pone.0280343)
Supplement: S1 Checklist — (DOCX) [file pone.0280343.s001.docx]

Adapted STROBE Checklist for Reporting EMA Studies (CREMAS)

| Title |  |  |
| --- | --- | --- |
| 1. Title | Include ecological momentary assessment in title and key words | Intensive longitutinal study used |
| Introduction |  |  |
| 2. Rationale | Briefly introduce the concept of EMA and provide reasons for utilizing EMA for this study or topic of interests | Within-person patterns using ILS method in final paragraph with aims and objectives |
| Methods |  |  |
| 3. Training | Indicate if, and by what methods, training of participants for EMA protocol was used | *Recruitment & enrolment*: download and instructions.  *Study design* describes pilot |
| 4. Technology | Describe what technology, if any, was used. Include the following information: device (eg, mobile phone, portable computer), model (eg, Nexus 4, iPod), operating system (eg, Android, Windows), and EMA program name | *Smartphone app* section  Also *Activity Sensor* section |
| 5. Wave Duration | State the number of waves for the study (eg, 2 monitoring periods over the course of 1 year) | Single wave only. |
| 6. Monitoring period | State the number of days each wave of the study lasted, and how many weekdays versus weekend days | *Smartphone app* (para3). Continuous days so not relevant to describe weekends separately. |
| 7. Prompting design | Indicate the prompting strategy used for the study (eg, event-based, interval-based, or a combination of the two). If using interval-based strategy, indicate what type of schedule is used (eg, fixed, random, or hybrid interval) | *Smartphone* app section |
| 8. Prompt Frequency | Intended frequency of prompts per day. Break down by weekdays and weekend days if applicable | *Smartphone* app section |
| 9. Design features | Describe any design feature to address potential sources of bias (eg, reactivity) or participant burden (eg, EMA questions appearing in different orders) | *Smartphone* app section – 3^rd^ paragraph – run-in period |
| Results |  |  |
| 10. Attrition | Indicate participant attrition throughout the study; report attrition rates both by monitoring days and waves, if applicable | *Completeness of data* |
| 11. Prompt delivery | Report number of EMA prompts that were planned to be delivered. If possible, also report the number of EMA prompts that were actually received by participants and indicate reasons for why prompts were not sent out (eg, technical issues or participant noncompliance reason such as phone was powered off) | *Methods: sample size* |
| 12. Latency | Report the amount of time from prompt signal to answering of prompt | Completeness of data / supplementary T5 |
| 13. Compliance rate | Report total answered EMA prompts across all subjects and the average number of EMA prompts answered per person. Report compliance rate both by monitoring days and waves, if applicable. Indicate reasons for noncompliance, if known | Completeness of data / supplementary T5 |
| 14. Missing data | Report whether EMA compliance is related to demographic or time-varying variables | Completeness of data – no association with mean fatigue |
| Discussion |  |  |
| 15. Limitations | Discuss limitations of the study, taking into account sources of potential bias when using EMA methods (eg, reactivity, use of technology) | Discussion |
| 16. Conclusions | Provide a general interpretation of results and discuss the benefits of using EMA | Discussion |
