# Supplementary material for: Within and between-day variation and associations of symptoms in Long Covid: Intensive longitudinal study
Source: PLoS One. 2023 Jan 19;18(1):e0280343. doi: 10.1371/journal.pone.0280343 (PMC9851560; doi:10.1371/journal.pone.0280343)
Supplement: S1 Fig — (DOCX) [file pone.0280343.s002.docx]

## S1 Fig. Relationship between correlation coefficient between symptoms and the mean VAS score on which correlation is based.


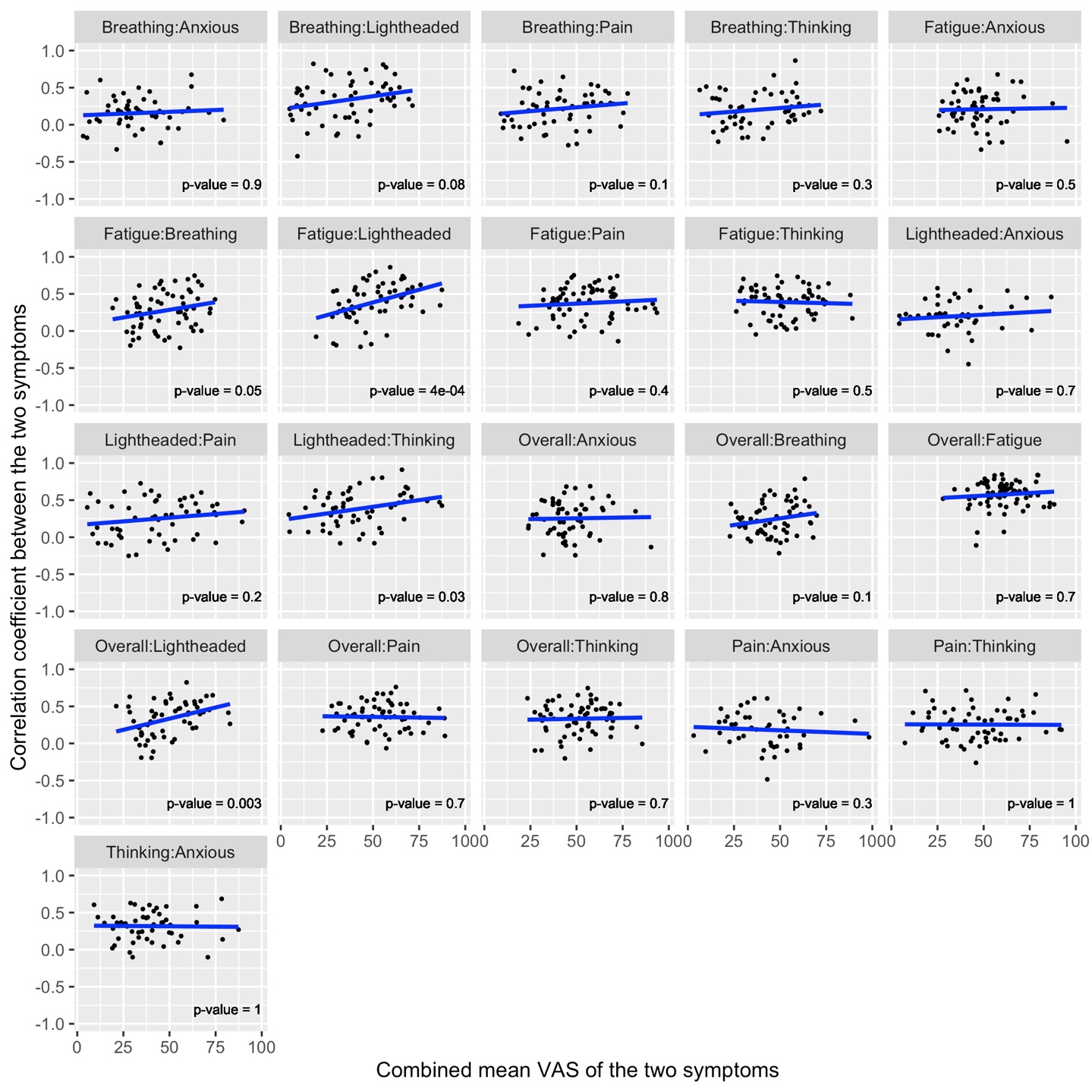


Each data point relates to one participant.
