# Supplementary material for: Within and between-day variation and associations of symptoms in Long Covid: Intensive longitudinal study
Source: PLoS One. 2023 Jan 19;18(1):e0280343. doi: 10.1371/journal.pone.0280343 (PMC9851560; doi:10.1371/journal.pone.0280343)
Supplement: S2 Fig — (DOCX) [file pone.0280343.s003.docx]

##

## S2 Fig: Heatmap of unadjusted correlations between symptoms at the individual participant level.


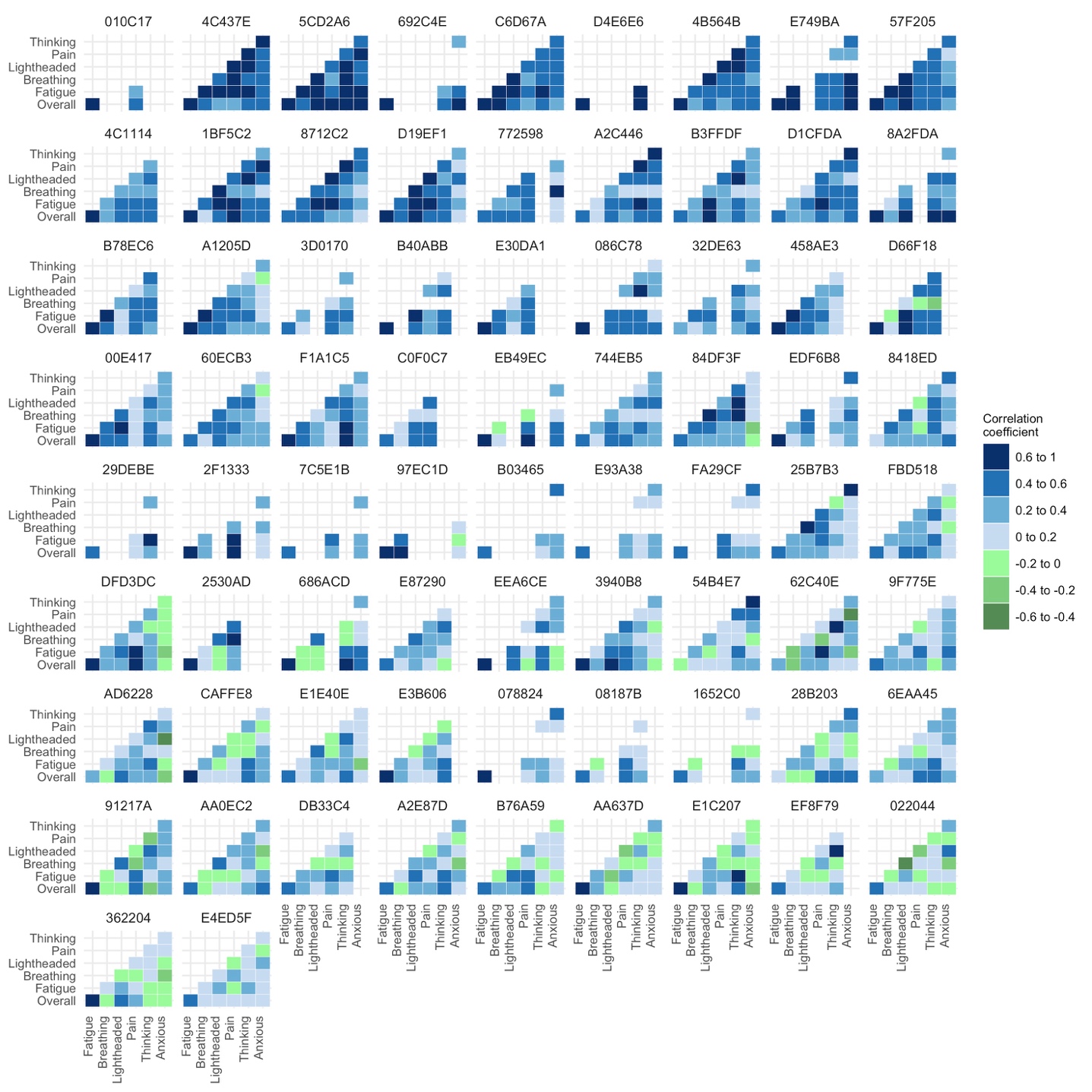


Figure includes all participants. Empty data cells indicate insufficient data to calculate a meaningful correlation.
