# Supplementary material for: Within and between-day variation and associations of symptoms in Long Covid: Intensive longitudinal study
Source: PLoS One. 2023 Jan 19;18(1):e0280343. doi: 10.1371/journal.pone.0280343 (PMC9851560; doi:10.1371/journal.pone.0280343)
Supplement: S3 Fig — (DOCX) [file pone.0280343.s004.docx]

## S3 Fig: Distribution of within-person correlations between objective activity from accelerometer and subjective measures of overall unwellness and demand (physical alone or combined physical, cognitive and emotional).


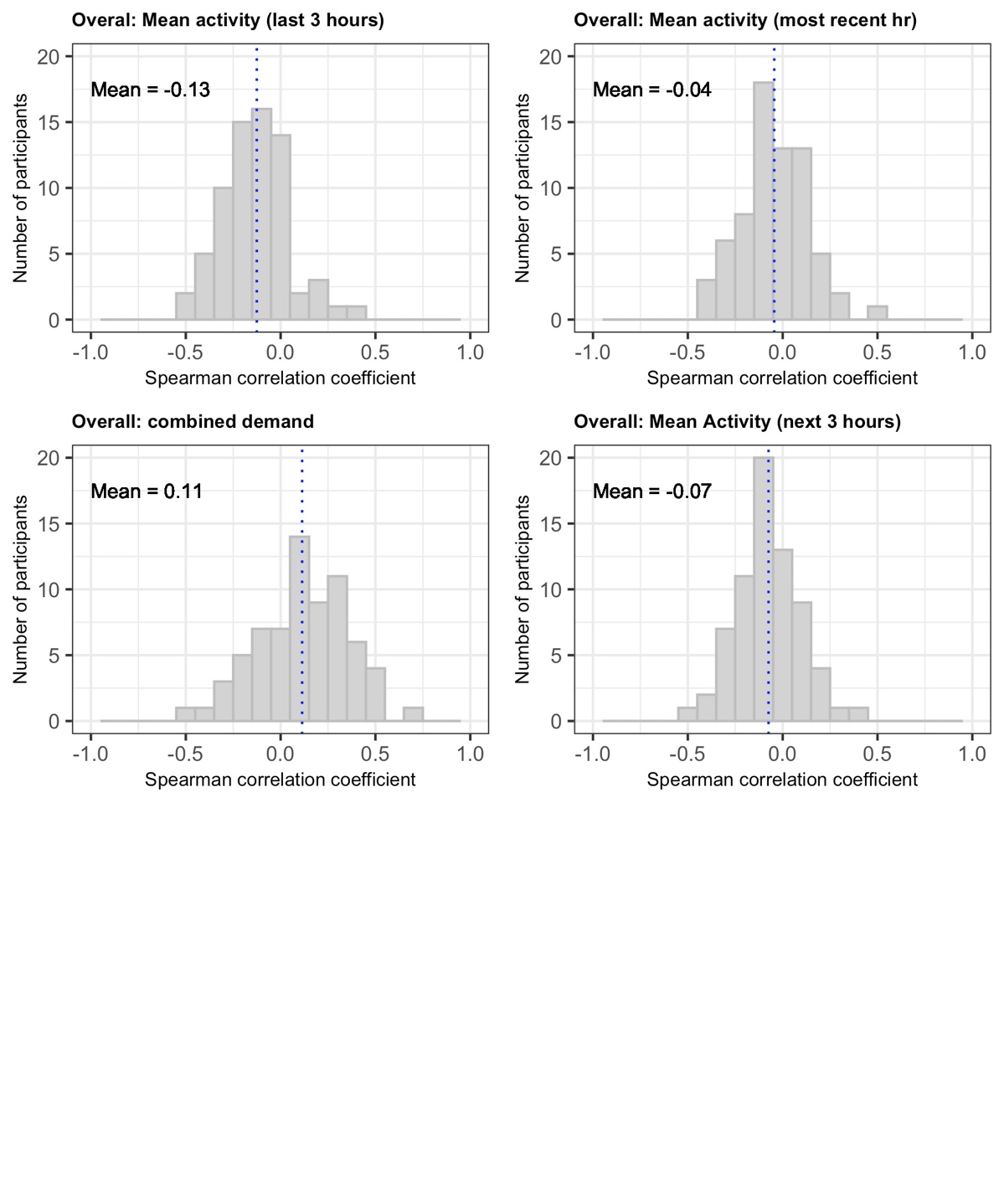


All activity measures refer to the 3 hour period before app data entry unless stated.
