# Supplementary material for: Within and between-day variation and associations of symptoms in Long Covid: Intensive longitudinal study
Source: PLoS One. 2023 Jan 19;18(1):e0280343. doi: 10.1371/journal.pone.0280343 (PMC9851560; doi:10.1371/journal.pone.0280343)
Supplement: S1 Table — (DOCX) [file pone.0280343.s005.docx]

## S1 Table: Smartphone app questions

| **A. App questions reported in this analysis** | |
| --- | --- |
| 1. About how you feel right now |  |
| Overall, how well do you feel just now? | VAS 0-100^[[1]](#footnote-1)^ |
| 2. Thinking about what you have been doing in the last few hours ….^^[[2]](#footnote-2)^^ |  |
| How physically demanding was it? | Likert 4 items |
| How mentally demanding was it? | Likert 4 items |
| How emotionally demanding was it? | Likert 4 items |
| 3. Please rate your symptoms just now |  |
| Fatigue | VAS 0-100 |
| Breathlessness | VAS 0-100 |
| Lightheaded or unsteady | VAS 0-100 |
| Altered smell or taste | VAS 0-100 |
| Pain | VAS 0-100 |
| Other physical symptoms | VAS 0-100 |
| Difficulty thinking clearly | VAS 0-100 |
| Feeling anxious or worried | VAS 0-100 |
| **B. Additional app questions not reported in this analysis** | |
| 4.Thinking ahead for today …^^[[3]](#footnote-3)^^ |  |
| Are you feeling positive or negative about what you will be doing? | Numeric 1:7 |
| How much energy do you think you will need? | Numeric 1:7 |
| How much energy do you think you currently have? | VAS 0-100 |
| 5. About your sleep last night ^^[[4]](#footnote-4)^^ |  |
| How good/refreshing was your sleep last night? | VAS 1:100 |
| What time did you go to bed? | time |
| How quickly did you get to sleep? | categorical |
| What time did you (finally) wake up? | time |
| How many times did you wake through the night? | categorical |

1. Score reversed to provide measure of overall unwellness [↑](#footnote-ref-1)
2. Not asked in first entry of the day (replaced with sleep related items) [↑](#footnote-ref-2)
3. Not asked in 20.00 entry. [↑](#footnote-ref-3)
4. Only asked at first entry of the day [↑](#footnote-ref-4)
