# Supplementary material for: Within and between-day variation and associations of symptoms in Long Covid: Intensive longitudinal study
Source: PLoS One. 2023 Jan 19;18(1):e0280343. doi: 10.1371/journal.pone.0280343 (PMC9851560; doi:10.1371/journal.pone.0280343)
Supplement: S2 Table — (DOCX) [file pone.0280343.s006.docx]

## S2 Table: Data entries by time by included participant

|  | Data entry prompt | | | | |
| --- | --- | --- | --- | --- | --- |
| User ID | 08:00 | 11:00 | 14:00 | 17:00 | 20:00 |
| 00E417 | 12 | 14 | 14 | 9 | 12 |
| 010C17 | 12 | 12 | 13 | 12 | 12 |
| 022044 | 13 | 13 | 9 | 9 | 11 |
| 078824 | 14 | 13 | 14 | 11 | 9 |
| 08187B | 9 | 13 | 10 | 9 | 13 |
| 086C78 | 11 | 8 | 9 | 7 | 11 |
| 1652C0 | 14 | 14 | 13 | 14 | 13 |
| 1BF5C2 | 2 | 9 | 10 | 12 | 13 |
| 2530AD | 9 | 9 | 11 | 11 | 12 |
| 25B7B3 | 10 | 13 | 13 | 14 | 12 |
| 28B203 | 10 | 7 | 7 | 10 | 13 |
| 29DEBE | 14 | 12 | 13 | 14 | 14 |
| 2F1333 | 11 | 9 | 10 | 10 | 14 |
| 32DE63 | 12 | 7 | 9 | 12 | 14 |
| 362204 | 9 | 8 | 8 | 8 | 9 |
| 3940B8 | 10 | 10 | 9 | 9 | 10 |
| 3D0170 | 13 | 11 | 10 | 13 | 13 |
| 458AE3 | 0 | 8 | 10 | 14 | 13 |
| 4B564B | 14 | 10 | 9 | 12 | 8 |
| 4C1114 | 14 | 10 | 11 | 14 | 13 |
| 4C437E | 12 | 11 | 10 | 12 | 10 |
| 54B4E7 | 11 | 9 | 10 | 4 | 9 |
| 57F205 | 4 | 12 | 12 | 12 | 10 |
| 5CD2A6 | 10 | 13 | 12 | 11 | 12 |
| 60ECB3 | 11 | 6 | 9 | 7 | 12 |
| 62C40E | 8 | 11 | 10 | 11 | 13 |
| 686ACD | 10 | 9 | 11 | 10 | 10 |
| 692C4E | 11 | 11 | 12 | 12 | 12 |
| 6EAA45 | 11 | 11 | 9 | 14 | 12 |
| 744EB5 | 6 | 9 | 7 | 8 | 9 |
| 772598 | 5 | 11 | 12 | 11 | 13 |
| 7C5E1B | 9 | 13 | 13 | 13 | 11 |
| 8418ED | 13 | 13 | 12 | 11 | 12 |
| 84DF3F | 10 | 13 | 11 | 13 | 13 |
| 8712C2 | 9 | 11 | 11 | 13 | 12 |
| 8A2FDA | 5 | 10 | 8 | 11 | 10 |
| 91217A | 3 | 9 | 9 | 9 | 6 |
| 97EC1D | 6 | 9 | 10 | 8 | 10 |
| 9F775E | 13 | 11 | 9 | 11 | 12 |
| A1205D | 13 | 14 | 12 | 13 | 12 |
| A2C446 | 8 | 12 | 8 | 10 | 6 |
| A2E87D | 12 | 14 | 14 | 14 | 14 |
| AA0EC2 | 5 | 13 | 12 | 13 | 14 |
| AA637D | 8 | 14 | 10 | 14 | 14 |
| AD6228 | 13 | 14 | 11 | 9 | 11 |
| B03465 | 13 | 14 | 14 | 14 | 14 |
| B3FFDF | 11 | 11 | 12 | 12 | 11 |
| B40ABB | 7 | 13 | 13 | 13 | 3 |
| B76A59 | 11 | 11 | 13 | 12 | 13 |
| B78EC6 | 13 | 7 | 10 | 5 | 9 |
| C0F0C7 | 8 | 7 | 9 | 7 | 10 |
| C6D67A | 11 | 6 | 10 | 8 | 10 |
| CAFFE8 | 3 | 13 | 8 | 7 | 8 |
| D19EF1 | 11 | 11 | 10 | 8 | 13 |
| D1CFDA | 8 | 14 | 14 | 14 | 14 |
| D4E6E6 | 13 | 11 | 12 | 12 | 8 |
| D66F18 | 12 | 12 | 13 | 9 | 9 |
| DB33C4 | 12 | 14 | 12 | 14 | 14 |
| DFD3DC | 1 | 11 | 11 | 10 | 13 |
| E1C207 | 14 | 13 | 13 | 12 | 13 |
| E1E40E | 10 | 11 | 14 | 14 | 13 |
| E30DA1 | 7 | 10 | 9 | 9 | 12 |
| E3B606 | 5 | 7 | 10 | 9 | 8 |
| E4ED5F | 3 | 8 | 9 | 9 | 11 |
| E749BA | 14 | 12 | 12 | 12 | 14 |
| E87290 | 14 | 14 | 14 | 13 | 14 |
| E93A38 | 14 | 14 | 14 | 13 | 13 |
| EB49EC | 14 | 13 | 13 | 13 | 13 |
| EDF6B8 | 13 | 14 | 14 | 13 | 14 |
| EEA6CE | 4 | 7 | 9 | 10 | 9 |
| EF8F79 | 12 | 13 | 12 | 13 | 14 |
| F1A1C5 | 9 | 10 | 8 | 6 | 11 |
| FA29CF | 13 | 10 | 11 | 11 | 11 |
| FBD518 | 13 | 9 | 14 | 13 | 14 |
