# Supplementary material for: Within and between-day variation and associations of symptoms in Long Covid: Intensive longitudinal study
Source: PLoS One. 2023 Jan 19;18(1):e0280343. doi: 10.1371/journal.pone.0280343 (PMC9851560; doi:10.1371/journal.pone.0280343)
Supplement: S3 Table — (DOCX) [file pone.0280343.s007.docx]

## S3 Table: Promptness of data entry by time of day.

|  | Data entry prompt | | | | |
| --- | --- | --- | --- | --- | --- |
| Delay | 08:00 | 11:00 | 14:00 | 17:00 | 20:00 |
| 0-10 mins | 349 | 447 | 454 | 463 | 468 |
| 11-35 mins | 251 | 266 | 276 | 252 | 272 |
| >35 mins | 132 | 100 | 83 | 98 | 111 |
| Total | 732 | 813 | 813 | 813 | 851 |
|  |  |  |  |  |  |
|  | Data entry prompt | | | | |
|  | 08:00 | 11:00 | 14:00 | 17:00 | 20:00 |
| 0-10 mins | 47.7% | 55.0% | 55.8% | 56.9% | 55.0% |
| 11-35 mins | 34.3% | 32.7% | 33.9% | 31.0% | 32.0% |
| >35 mins | 18.0% | 12.3% | 10.2% | 12.1% | 13.0% |
|  |  |  |  |  |  |
