# Supplementary material for: Within and between-day variation and associations of symptoms in Long Covid: Intensive longitudinal study
Source: PLoS One. 2023 Jan 19;18(1):e0280343. doi: 10.1371/journal.pone.0280343 (PMC9851560; doi:10.1371/journal.pone.0280343)
Supplement: S1 Text — (DOCX) [file pone.0280343.s008.docx]

# Supporting information

## S1. File: Embodied Predictive Interoceptive Coding

Lay models of symptom perception work in a “bottom-up” fashion. In this, the brain is seen as collecting incoming nerve signals from multiple sources, then integrating them to create a percept (or image) of the body or environment. This would be computationally extremely inefficient and appears not to be what actually happens. Instead the brain uses a more efficient top-down model in which it automatically (non-consciously) supplies a set of prior probabilities (or predictions) of the current state and then compares these with incoming signals and makes changes in order to continuously update percepts. Any inconsistency between predictions and incoming signals is resolved by minimising the difference (or “prediction error”) between the two. This form of Bayesian inference is referred to as predictive coding (and sometimes as the “Bayesian Brain”) [24]. It is important to emphasise that these predictions are not conscious expectations but non-conscious prior probabilities generated by the brain based on previous patterns. While they can be cued by factors such as a particular environment or behaviour, they cannot be consciously changed.

Embodied Predictive Interoceptive Coding (EPIC) [23] is a widely-used model for understanding bodily experience and symptoms based on this predictive coding model. It relates to interoception, the brain’s non-conscious sensing, interpreting and reacting to the states of the body [22]. To date, EPIC models have been investigated across a range of conditions [54] and symptoms that include fatigue, [58] breathlessness,[53], pain, [59] and dizziness [60].

According to EPIC, when prior probabilities are weak or uncertain and the incoming signals are strong then percepts will be dominated by the incoming signals. However when incoming signals are weak relative to priors, then percepts will be dominated by priors. This is relevant to persistent physical symptoms after acute illness because (a) as body organ pathology resolves the incoming signals are likely to diminish (b) the neural processes of interoception may be affected following Covid19, thus reducing the accuracy of incoming signals (c) as priors are consolidated by learning, the longer that symptoms percepts are dominated by priors, the stronger those priors become.
